# ZIF-8-Confined and APTES-Passivated CsPbBr_3_ Quantum Dots for Fluorescence Detection of Enrofloxacin Residues in Foods

**DOI:** 10.3390/foods15162772

**Published:** 2026-08-07

**Authors:** Guanru Yi, Shaoyu Lv, Longwei Fu, Zhixiang Xu, Shiyong Wang, Longhua Xu

**Affiliations:** College of Food Science and Engineering, Shandong Agricultural University, Tai’an 271018, China; yi784904607@163.com (G.Y.); lvshaoyu2021@163.com (S.L.); 18863015339@163.com (L.F.); zhixiangxu@sina.com (Z.X.); hzwangshiyong@163.com (S.W.)

**Keywords:** enrofloxacin, food safety, fluorescence sensing, antibiotic residues

## Abstract

Enrofloxacin residues in foods pose potential risks to food safety and public health, creating demand for rapid, sensitive, and matrix-compatible screening methods. All-inorganic CsPbBr_3_ quantum dots (QDs) are promising fluorescent probes because of their strong photoluminescence, but their poor stability in polar media greatly restricts their practical use in food analysis. Herein, ZIF-8-confined and APTES-passivated CsPbBr_3_ QDs were developed as an ethanol-compatible fluorescent sensor for enrofloxacin detection in foods. Unlike reported CsPbBr_3_@ZIF-8 systems mainly used for metal-ion sensing in aqueous media, this work integrates ZIF-8-confined in-situ growth with APTES-assisted silane passivation to improve the stability and applicability of CsPbBr_3_ QDs in ethanol-based food extracts. ZIF-8 provided a confined microenvironment, while the APTES-derived silane layer further enhanced fluorescence stability. The sensor exhibited a distinct turn-off fluorescence response toward enrofloxacin, with a linear range of 0.1–25.0 mg·L^−1^ and a limit of detection of 0.058 mg·L^−1^, mainly attributed to electron transfer with CsPbBr_3_@ZIF-8. The method was successfully applied to honey, milk, fish, and shrimp samples, achieving recoveries of 85.2–106.8%. Results obtained for aquatic products showed no significant difference from HPLC analysis (*p* > 0.05), demonstrating the potential of this sensor for rapid enrofloxacin residue screening in complex food matrices.

## 1. Introduction

Enrofloxacin (ENR), a third-generation quinolone antibiotic, exhibits broad-spectrum antibacterial activity against Gram-positive bacteria, Gram-negative bacteria, and mycoplasma [1]. By inhibiting bacterial DNA replication, ENR has been widely used in animal husbandry and aquaculture owing to its strong tissue penetration and favorable pharmacokinetic stability [2]. However, the excessive or inappropriate use of ENR can result in residue accumulation in edible animal tissues [3], thereby increasing the likelihood of human exposure via animal-derived foods and raising concerns regarding neurotoxicity, allergic responses, and the development of antimicrobial resistance [4]. Therefore, monitoring ENR residues in animal-derived foods is of great significance for food safety and public health. Notably, ENR can be metabolized to ciprofloxacin (CIP) in vivo, and the sum of ENR and CIP is commonly used as the marker residue for regulatory surveillance. Therefore, selective ENR determination is useful for targeted preliminary screening, although chromatographic confirmation of ENR and CIP remains necessary for regulatory assessment. According to relevant food safety regulations, ENR-related residues in animal-derived foods are regulated at the μg kg^−1^ level. For example, the maximum residue limit for enrofloxacin-related residues is commonly set at 100 μg kg^−1^ in milk and fish muscle, indicating that analytical methods should be evaluated with reference to this residue level. To date, various analytical methods, including electrochemical analysis [5], capillary electrophoresis [6], surface plasmon resonance [7], and chemiluminescence [8], have been applied for ENR detection. Nevertheless, these approaches are still constrained by several drawbacks, including the requirement for costly analytical equipment, labor-intensive sample preparation, limited operational stability, and relatively low analytical efficiency [9,10]. Consequently, developing a simple, rapid, and efficient method for ENR detection in food matrices remains highly desirable.

Perovskite quantum dots (PQDs), especially CsPbBr_3_ nanocrystals, provide an attractive fluorescent platform for analytical sensing because of their intense emission, narrow photoluminescence band, high defect tolerance, and facile preparation under mild conditions [11]. Compared with conventional semiconductor quantum dots, CsPbBr_3_ QDs can be obtained rapidly through room-temperature ligand-assisted reprecipitation, avoiding harsh synthetic conditions and enabling their use in rapid sensing systems [12,13]. These optical and synthetic advantages have promoted their applications in the determination of metal ions, pesticides, food additives, and antibiotic residues [14]. Nevertheless, the ionic nature and low formation energy of CsPbBr_3_ QDs make them vulnerable to moisture, oxygen, heat, and polar solvents [15]. This instability often causes fluorescence attenuation and structural degradation, which seriously limits their application in food safety analysis, where polar extraction media and complex sample matrices are commonly involved.

To improve the environmental robustness of PQDs, several modification strategies have been explored, such as ion doping, ligand regulation, surface passivation, and matrix encapsulation [16,17,18]. Among these approaches, introducing a porous host is particularly useful because it can spatially confine the growth of PQDs and isolate them from external quenchers. Metal–organic frameworks (MOFs), constructed from metal nodes and organic linkers, possess ordered porosity, large surface area, and abundant interaction sites, making them suitable hosts for stabilizing luminescent nanomaterials [19]. ZIF-8, a representative zeolitic imidazolate framework, is especially suitable for this purpose because it can be synthesized under mild conditions and has a stable porous structure. Its sodalite-type cages and narrow apertures can create restricted microenvironments for the nucleation and growth of CsPbBr_3_ nanocrystals, thereby suppressing particle aggregation and enhancing resistance to polar media [20].

Previous studies have shown that coupling fluorescent nanomaterials with ZIF-8 can improve their stability, adsorption capacity, and sensing performance [21], which may be attributed to the porous framework and abundant surface sites of ZIF-8. These features may facilitate the local enrichment of fluoroquinolone antibiotics through hydrogen bonding, electrostatic attraction, coordination interactions, and π–π interactions [22]. Constructing CsPbBr_3_@ZIF-8 nanocomposites is therefore a feasible route to combine the strong fluorescence of CsPbBr_3_ QDs with the protective and enrichment functions of ZIF-8. However, reported CsPbBr_3_@ZIF-8 systems have mainly focused on enhancing water stability and detecting metal ions in aqueous media, whereas their use for antibiotic residue detection in complex food matrices remains insufficiently explored. In particular, maintaining stable fluorescence in ethanol-based food extracts is still a critical challenge for practical food analysis.

In this study, ZIF-8-confined and APTES-passivated CsPbBr_3_ QDs were developed as a turn-off fluorescent sensor for the detection of ENR residues in foods. CsPbBr_3_ QDs were generated in situ within the porous ZIF-8 framework at room temperature, while APTES-assisted silane passivation was introduced to further improve the fluorescence stability of the composite in ethanol. The ZIF-8 framework provided confined protection for CsPbBr_3_ QDs and potential adsorption sites for ENR enrichment, whereas the APTES-derived silane layer reduced environmental interference in the sensing medium. The fluorescence response, selectivity, and quenching mechanism were systematically investigated, and the applicability of the method was verified in honey, milk, fish, and shrimp samples, with HPLC comparison performed for aquatic products. This work provides an ethanol-compatible perovskite-MOF fluorescent platform for rapid ENR residue screening in complex food matrices.

## 2. Experimental Section

### 2.1. Chemicals and Reagents

Zinc nitrate hexahydrate (Zn(NO_3_)_2_·6H_2_O, ≥99%) and 2-methylimidazole (≥99%) were purchased from Tianjin Kemiou Chemical Reagent Co., Ltd. (Tianjin, China) Lead bromide (PbBr_2_, 99%), tetra-n-octylammonium bromide (TOAB, 98%), n-octanoic acid (99%), and 3-aminopropyltriethoxysilane (APTES, 99%) were supplied by Aladdin Biochemical Technology Co., Ltd. (Shanghai, China). Cesium carbonate (Cs_2_CO_3_, 99%), enrofloxacin (ENR, 98%), norfloxacin (NOR, 98%), ciprofloxacin (CIP, 98%), ofloxacin (OFL, 98%), levofloxacin (LEV, 98%), pefloxacin (PEF, 98%), fleroxacin (FLX, 98%), and tetracycline (TC, 98%) were supplied by Shanghai Macklin Biochemical Co., Ltd. (Shanghai, China) Toluene (≥99.5%), methanol (≥99.5%), ethanol (≥99.7%), dichloromethane (≥99.5%), acetonitrile, n-hexane (98%), and N,N-dimethylformamide (DMF, ≥99.5%) were purchased from Tianjin Kaitong Chemical Reagent Co., Ltd. (Tianjin, China). All chemicals were used as received without further purification.

### 2.2. Instruments

The optical properties of the samples were evaluated by fluorescence and UV-vis absorption measurements. Fluorescence emission spectra were collected on an LF-1701009 fluorescence-phosphorescence spectrophotometer (Thermo, Madison, WI, USA), while UV-vis absorption spectra were obtained with a UV-3600 Plus UV-vis-NIR spectrophotometer (Shimadzu, Kyoto, Japan). The morphology and microstructural features of the materials were examined with a GEMINI 300 field-emission scanning electron microscope (ZEISS, Jena, Germany) and a TALOS F200S transmission electron microscope (FEI, Hillsboro, OR, USA), respectively. The surface elemental composition and chemical states were analyzed by X-ray photoelectron spectroscopy using an ESCALAB 250Xi spectrometer (Thermo, East Grinstead, UK). Fluorescence decay curves were measured using an FLS980 fluorescence spectrometer (Edinburgh Instruments Ltd., Livingston, UK) with a pulsed excitation source operating at 365 nm. The emission decay was recorded at 520 nm.

### 2.3. Synthesis of ZIF-8

ZIF-8 was synthesized according to a reported method with slight modification [23]. In detail, 1.23 mmol of Zn(NO_3_)_2_·6H_2_O and 9.88 mmol of 2-methylimidazole were separately dissolved in 25 mL of methanol. The two solutions were then mixed under magnetic stirring at room temperature until the mixture became turbid, followed by aging for 1 h. The resulting white dispersion was centrifuged at 11,200× *g* for 10 min to collect the ZIF-8 nanocrystals. Finally, the product was washed with methanol and dried at 50 °C in a vacuum oven.

### 2.4. Synthesis of CsPbBr_3_@ZIF-8

The cesium precursor was prepared by dissolving 0.5 mmol of Cs_2_CO_3_ in 5.0 mL of n-octanoic acid under magnetic stirring. Then, 0.5 mmol of PbBr_2_ and the required amount of TOAB were dissolved in 5.0 mL of toluene under continuous stirring. After a clear solution was obtained, 100 mg of ZIF-8 powder was added and stirred for 30 min. Subsequently, 0.5 mL of the cesium precursor solution and 0.4 mL of APTES were added to the above suspension. After stirring for another 30 min at room temperature, the product was collected by centrifugation at 7155× *g* for 10 min, washed with ethanol, and dried at 50 °C in a vacuum oven to obtain CsPbBr_3_@ZIF-8.

### 2.5. Food Sample Preparation

To comprehensively assess the practical applicability of CsPbBr_3_@ZIF-8 for ENR detection, four representative food matrices, including honey, milk, fish, and shrimp, were selected to represent sugar-rich and protein/lipid-rich matrices. Honey and milk samples were pretreated following previously reported methods [24,25], respectively, with minor modifications. Fish and shrimp samples (10.0 g) were processed using a slightly modified procedure based on GB 31656.3-2021 [26], which was later superseded by GB 31656.3-2025 [27] without substantive changes to the relevant sample preparation procedure. The final extracts were reconstituted in 2.0 mL of ethanol for fluorescence detection.

A 4.0 g honey sample was diluted with 8.0 mL of deionized water, followed by shaking for 5 min. Subsequently, 20.0 mL of dichloromethane was added, and the mixture was homogenized by sonication and shaking. The resulting mixture was centrifuged at 2800× *g* for 5 min to separate the organic phase, which was then collected and evaporated to dryness under a nitrogen stream at 50 °C. The dried residue was reconstituted in 2.0 mL of ethanol and used for fluorescence detection.

For milk samples, 4.0 mL of milk was transferred into a centrifuge tube, and 12.0 mL of acetonitrile was added to precipitate proteins and extract the analyte. The mixture was vortexed for 30 s, sonicated for 30 min, and then centrifuged at 2400× *g* for 15 min at room temperature. The supernatant was collected and mixed with 12.0 mL of n-hexane for lipid removal. After phase separation, the n-hexane layer was discarded, and the defatting procedure was repeated twice. The resulting extract was filtered, evaporated to dryness at 50 °C using an EYELA N-1300 rotary evaporator (Tokyo Rikakikai Co., Ltd., Tokyo, Japan). The dried residue was then reconstituted in 2.0 mL of ethanol for fluorescence detection.

### 2.6. FL Sensing Detection of ENR

CsPbBr_3_@ZIF-8 powder (2.0 mg) was dispersed in 2.0 mL of the ENR standard solution or the final sample extract, thoroughly mixed, and incubated in the dark at room temperature for exactly 6 min, after which the fluorescence intensity was recorded immediately. To systematically eliminate the hydrolytic degradation of the perovskite lattice and control protonation-induced variations, all antibiotic working solutions were strictly prepared in anhydrous ethanol, thereby maintaining a non-aqueous environment with consistent solvent-solute interactions across all comparative testing groups. The fluorescence signal was then measured using a fluorescence–phosphorescence spectrophotometer. The fluorescence emission was recorded under excitation at 365 nm, with excitation/emission slit width of 5 nm and a photomultiplier tube voltage of 500 V. The fluorescence intensity at 520 nm was used for quantitative analysis. All measurements were performed in triplicate. All measurements were performed using three independent experiments, and the average values were used for quantitative analysis. Error bars represent the standard deviations of three independent measurements.

### 2.7. HPLC Method Validation

To verify the accuracy and applicability of the proposed fluorescence sensing method, the results obtained for real food samples were cross-validated using an HPLC reference method. Detailed matrix-specific sample-preparation procedures are provided in Supplementary Method S1.

The HPLC system (Shimadzu, Japan) was equipped with a fluorescence detector operating at excitation and emission wavelengths of 280 and 480 nm, respectively. Chromatographic separation was achieved using a C18 reversed-phase column (250 × 4.6 mm, 5 μm). The mobile phase consisted of 0.01 mol L^−1^ tetrabutylammonium bromide solution and acetonitrile (94:6, *v*/*v*), The column temperature was maintained at 30 °C, and the injection volume was 10 μL.

### 2.8. Greenness Assessment Methodology

The greenness of the proposed method was evaluated using the AGREE metric based on the 12 principles of green analytical chemistry. All principles were assigned equal weights, and the assessment covered sample preparation and fluorescence detection.

## 3. Results and Discussion

### 3.1. Characterization of CsPbBr_3_@ZIF-8

**Scheme 1:**
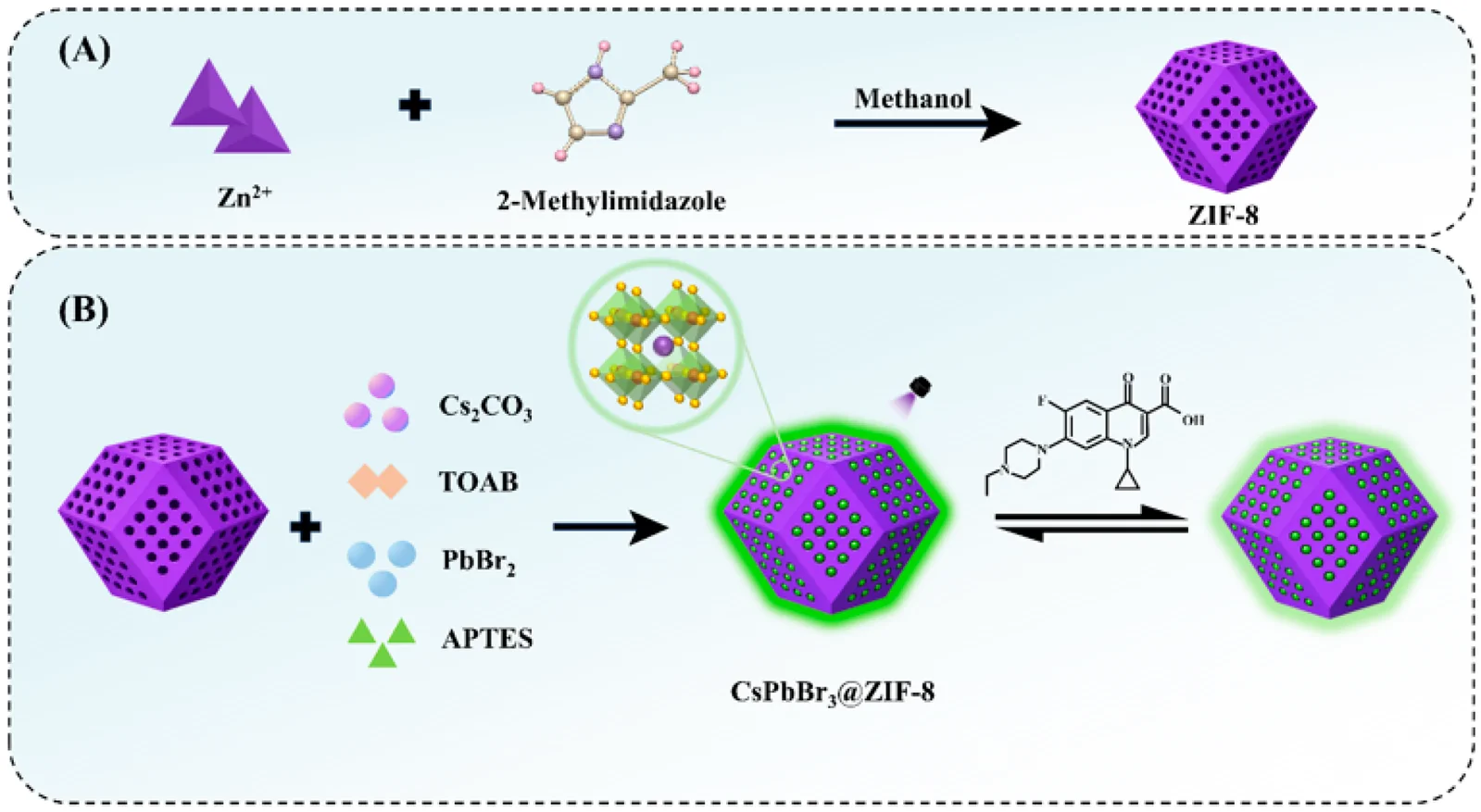
Synthesis of (**A**) ZIF-8 and (**B**) CsPbBr_3_@ZIF-8 for fluorescence sensing ENR.

CsPbBr_3_@ZIF-8 fluorescent nanocomposites were fabricated via a ligand-assisted reprecipitation strategy, in which the porous ZIF-8 framework provided a confined space for the in-situ growth of CsPbBr_3_ QDs, as illustrated in Scheme 1. Subsequently, APTES was hydrolyzed by trace moisture in air to form a silane-derived protective layer on the nanocomposite surface, thereby improving its stability. The morphology and physico chemical properties of CsPbBr_3_@ZIF-8 were systematically characterized by SEM, TEM, FT-IR, and XPS.

**Figure 1:**
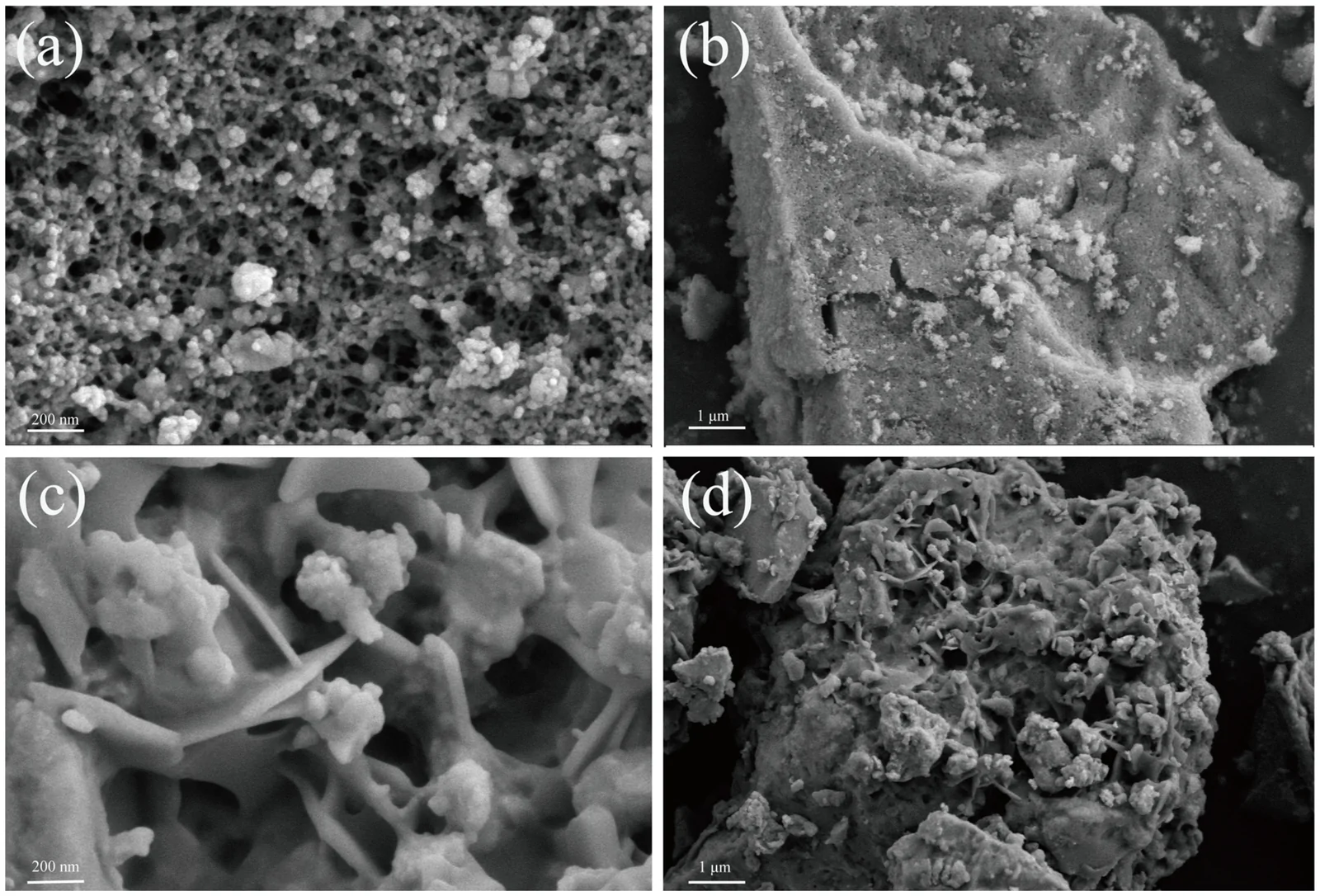
SEM images of ZIF-8 (**a**,**b**) and CsPbBr_3_@ZIF-8 (**c**,**d**).

**Figure 2:**
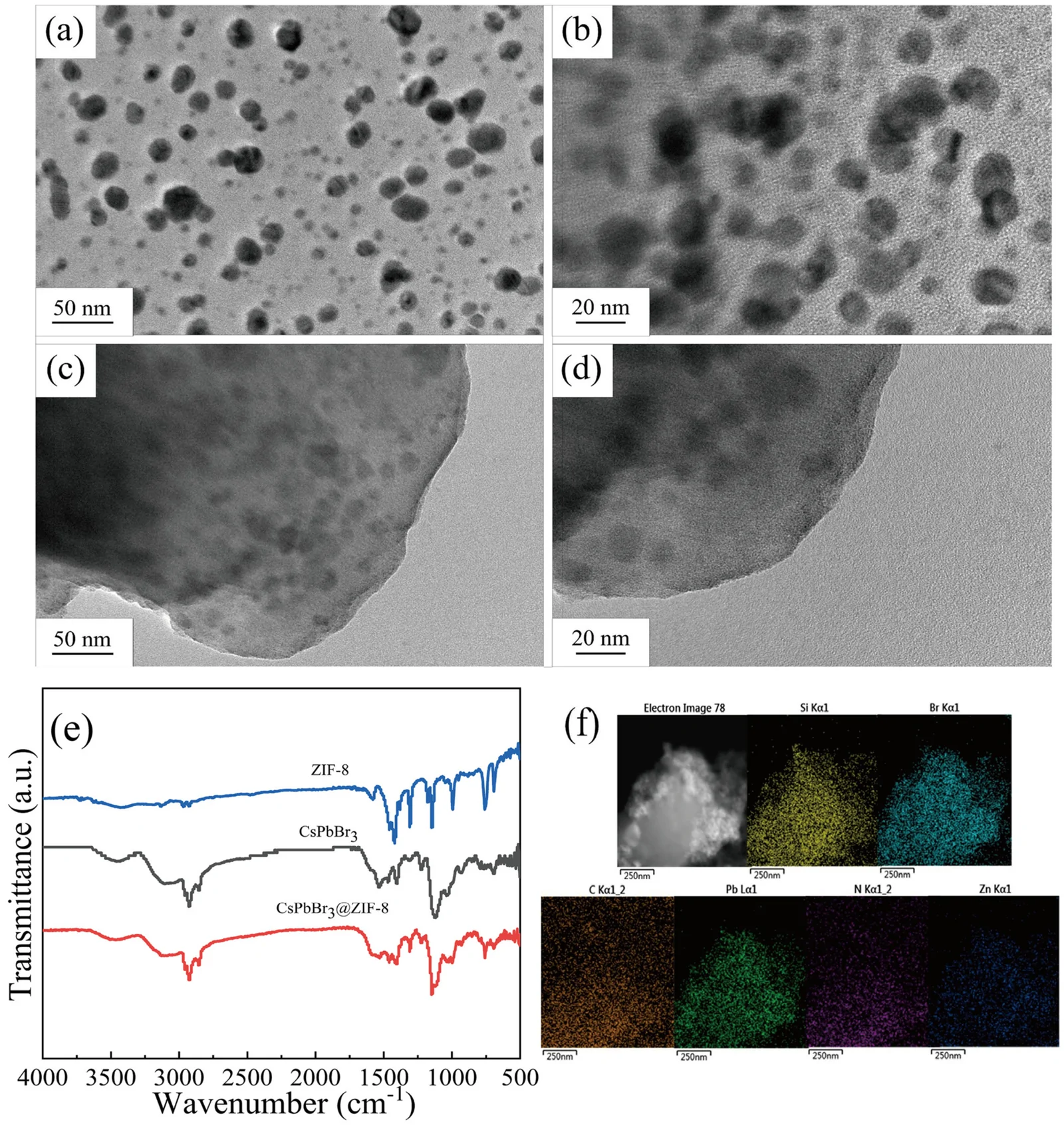
TEM images of (**a**,**b**) CsPbBr_3_ and (**c**,**d**) CsPbBr_3_@ZIF-8. (**e**) FT-IR spectra of ZIF-8, CsPbBr_3_, and CsPbBr_3_@ZIF-8. (**f**) Elemental mapping of CsPbBr_3_@ZIF-8.

The morphologies of ZIF-8 and CsPbBr_3_@ZIF-8 were characterized by SEM. As illustrated in Figure 1, ZIF-8 maintained its typical polyhedral structure after the incorporation of CsPbBr_3_, indicating that the in-situ growth of CsPbBr_3_ QDs did not destroy the ZIF-8 framework. After APTES modification, the composite exhibited a relatively smoother surface, which can be attributed to the hydrolysis and surface deposition of APTES. Since the CsPbBr_3_ QDs embedded in the ZIF-8 matrix could not be directly resolved from the SEM images, TEM characterization was further performed to observe the microstructure of CsPbBr_3_ and CsPbBr_3_@ZIF-8. As shown in Figure 2a,b, the CsPbBr_3_ QDs exhibited a nanoscale particulate morphology with relatively uniform dispersion. After incorporation into ZIF-8, nanoscale dark-contrast domains were observed within the ZIF-8 particles (Figure 2c,d). These domains showed dimensions and morphology comparable to those of the pristine CsPbBr_3_ QDs, and, together with the elemental mapping results, support the successful incorporation of CsPbBr_3_ into the ZIF-8 matrix.

FT-IR spectroscopy was employed to examine the functional groups and structural evolution of ZIF-8, CsPbBr_3_, and CsPbBr_3_@ZIF-8. As shown in Figure 2e, the characteristic vibration bands of ZIF-8, including those associated with the imidazole ring (758 and 1310 cm^−1^), were retained in CsPbBr_3_@ZIF-8, indicating that the ZIF-8 framework was preserved after the in-situ growth of CsPbBr_3_ QDs [28]. Compared with pristine CsPbBr_3_, the emergence of characteristic ZIF-8 signals in CsPbBr_3_@ZIF-8 further supports the successful integration of CsPbBr_3_ with the porous ZIF-8 matrix. Notably, the absorption band at 1125 cm^−1^, assigned to Si-O-Si stretching vibration, suggests the introduction of APTES-derived silane species [29]. In addition, the bands at approximately 2850 and 2920 cm^−1^ corresponded to C-H stretching vibrations, while the signals around 1540 and 3480 cm^−1^ were related to N-H vibrations from surface organic components. These results indicate the successful construction of ZIF-8-confined CsPbBr_3_ QDs and provide evidence for APTES-assisted silane passivation, which is beneficial for improving fluorescence stability in ethanol.

Elemental mapping was further performed to analyze the elemental distribution of CsPbBr_3_@ZIF-8. As illustrated in Figure 2f, the signals of Zn, C, and N were distributed throughout the selected region, confirming the presence of the ZIF-8 framework in the composite. Meanwhile, Pb and Br signals were clearly observed in the composite region and showed spatial association with the ZIF-8-related elements, suggesting the coexistence of CsPbBr_3_ and ZIF-8 within the composite structure. Although the Si signal was relatively weak in the EDX mapping, the introduction of APTES-derived silane species was supported by the FT-IR and XPS results. These elemental mapping results provide additional evidence for the successful construction of CsPbBr_3_@ZIF-8 and demonstrate the coexistence of ZIF-8, CsPbBr_3_, and the APTES-derived protective component within the composite.

**Figure 3:**
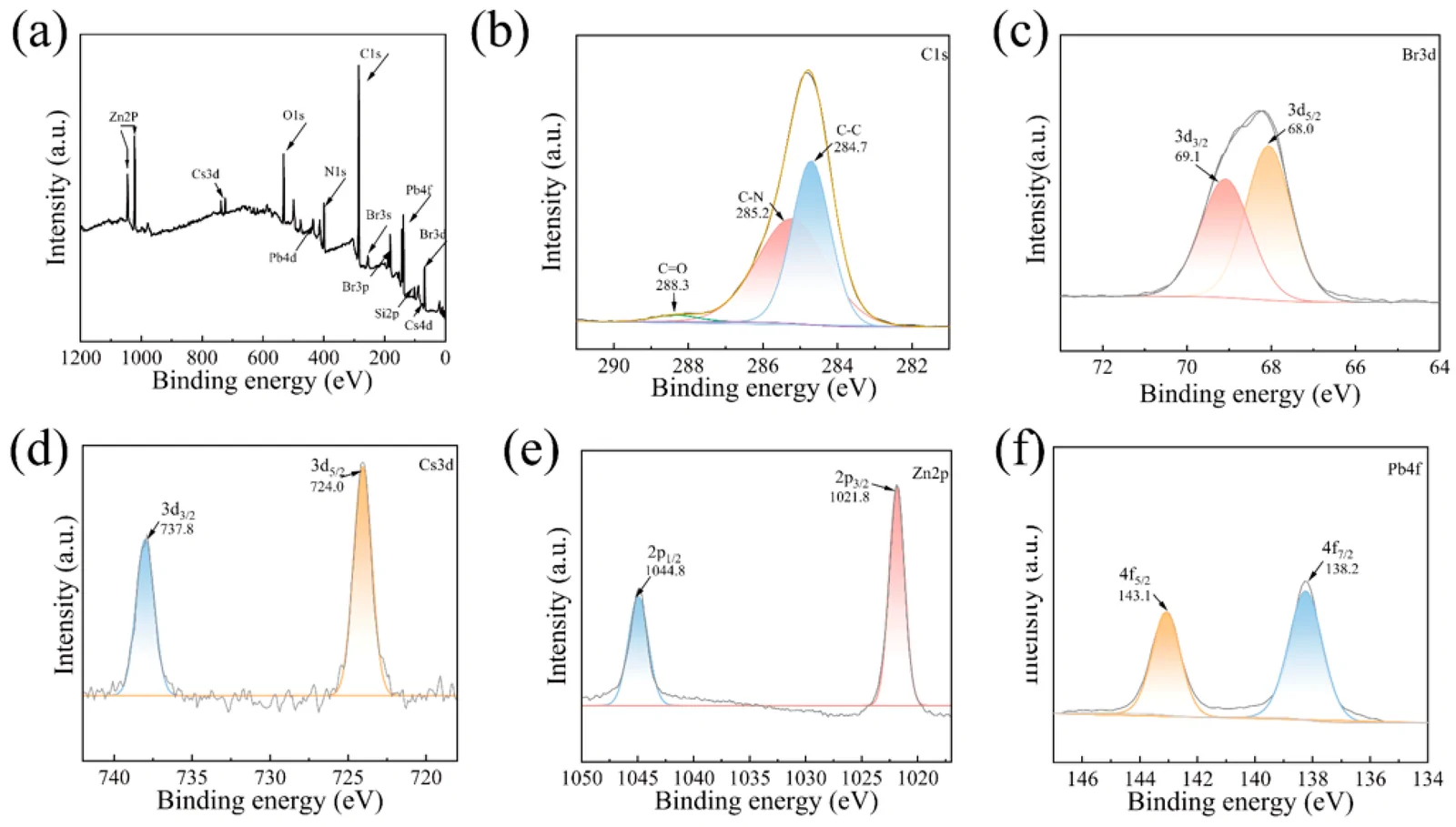
(**a**) XPS spectra of CsPbBr_3_@ZIF-8; High-resolution XPS spectra of C 1s (**b**), Br 3d (**c**), Cs 3d (**d**), Zn 2p (**e**) and Pb 4f (**f**).

XPS was employed to gain further insight into the elemental composition and chemical states of CsPbBr_3_@ZIF-8. As shown in Figure 3a, the XPS survey spectrum confirmed the expected elemental composition of the composite, with signals assignable to CsPbBr_3_, ZIF-8, and APTES-derived silicon-containing species [30]. The presence of the Si 2p signal at 102.38 eV can be assigned to Si-O-Si, further confirming the formation of the APTES-derived silica-like protective layer, which is consistent with the FT-IR results. In the high-resolution C 1s spectrum (Figure 3b), three fitted peaks were observed at 284.7, 285.2, and 288.3 eV, corresponding to C-C, C-N, and C=O bonds, respectively. The C=O signal is mainly associated with n-octanoic acid, while the C, N, and O signals originate from ZIF-8, APTES, and the organic ligands of CsPbBr_3_ [19]. In addition, the Br 3d spectrum displayed two peaks at 68.0 and 69.1 eV, corresponding to Br 3d_5_/_2_ and Br 3d_3_/_2_, respectively (Figure 3c). The Cs 3d spectrum showed two characteristic peaks at 724.0 and 737.8 eV, assigned to Cs 3d_5_/_2_ and Cs 3d_3_/_2_, respectively (Figure 3d). Moreover, the Zn 2p peaks at 1021.8 and 1044.8 eV confirmed the presence of the ZIF-8 framework (Figure 3e), while the Pb 4f peaks at 138.2 and 143.1 eV were attributed to Pb 4f_7_/_2_ and Pb 4f_5_/_2_, respectively (Figure 3f) [31]. These XPS results provide further evidence for the coexistence of the CsPbBr_3_-, ZIF-8-, and APTES-related species in the isolated composite and, together with the TEM and EDX results, support the formation of the CsPbBr_3_/ZIF-8 nanocomposite.

Taken together, the successful formation of the CsPbBr_3_/ZIF-8 nanocomposite was supported by the in-situ synthetic route and the complementary structural and compositional evidence. CsPbBr_3_ was generated in the presence of ZIF-8 rather than by simply mixing separately prepared CsPbBr_3_ QDs and ZIF-8 particles. TEM revealed CsPbBr_3_-like nanoscale high-contrast domains within the projected regions of the ZIF-8 particles, while STEM-EDX mapping showed the spatial association of Pb and Br with the Zn-, C-, and N-containing ZIF-8 regions. Together with the retention of the ZIF-8 framework and the introduction of APTES-derived siloxane species confirmed by FT-IR, as well as the coexistence and chemical states of the constituent species verified by XPS, these results collectively support the formation of the CsPbBr_3_/ZIF-8 nanocomposite rather than the independent presence of its individual components. A similar complementary characterization strategy involving electron microscopy, elemental mapping, FT-IR, and XPS has also been used to confirm the formation of related CsPbBr_3_@ZIF nanocomposites [32].

### 3.2. Luminescent Properties and Stability of CsPbBr_3_@ZIF-8

**Figure 4:**
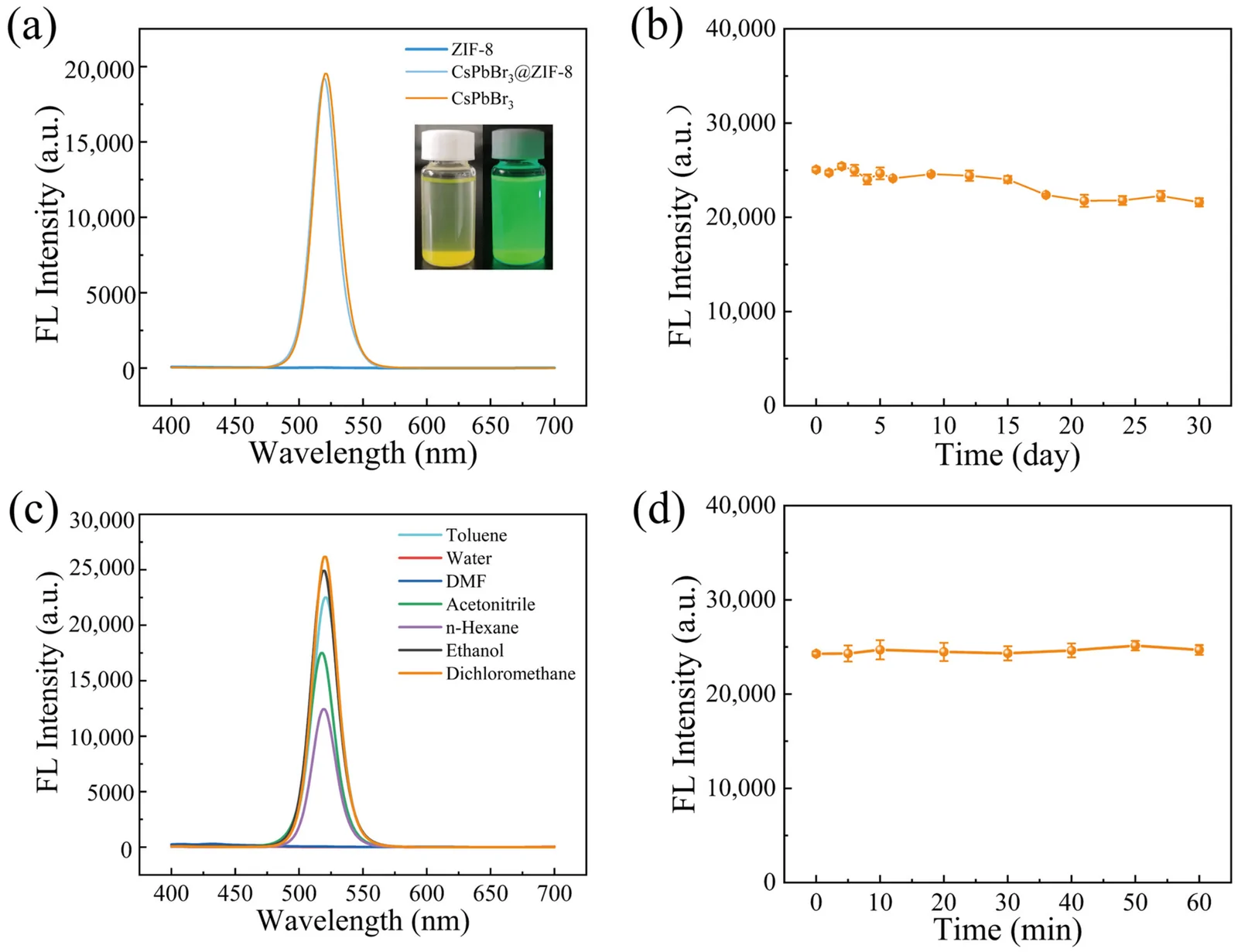
(**a**) Fluorescence emission spectra of ZIF-8, CsPbBr_3_ and CsPbBr_3_@ZIF-8 under 365 nm excitation; (**b**) Fluorescence intensity variation in CsPbBr_3_@ZIF-8 powder exposed to natural light for 1 month, with fluorescence measurements performed after dispersing the powder in ethanol; (**c**) Fluorescence spectra of CsPbBr_3_@ZIF-8 in different solvents, including n-hexane, toluene, dichloromethane, ethanol, acetonitrile, DMF, and water; (**d**) Fluorescence stability of CsPbBr_3_@ZIF-8 ethanol suspension within 1 h.

The fluorescence properties and storage stability of CsPbBr_3_@ZIF-8 were further evaluated. As shown in Figure 4a, CsPbBr_3_ exhibited a strong fluorescence emission peak at approximately 520 nm, while ZIF-8 showed almost no emission under the same excitation conditions. After incorporation into ZIF-8, CsPbBr_3_@ZIF-8 retained a similar emission profile, indicating that the fluorescence signal of the composite mainly originated from CsPbBr_3_ rather than the ZIF-8 framework. The inset photographs further show the bright green emission of CsPbBr_3_@ZIF-8 under UV light, which is consistent with the fluorescence spectrum. To evaluate its storage stability, CsPbBr_3_@ZIF-8 powder was stored under natural light for 30 days and periodically dispersed in ethanol for fluorescence measurement. As shown in Figure 4b, the initial fluorescence intensity of CsPbBr_3_@ZIF-8 was approximately 25,000 a.u. During the first 15 days of storage, the fluorescence intensity fluctuated slightly and remained at approximately 95%–100% of its initial value, indicating good short-term stability. After 30 days, approximately 83% of the initial fluorescence intensity was retained, demonstrating the favorable storage stability of CsPbBr_3_@ZIF-8. The enhanced stability can be attributed to the confinement and protection effects of the ZIF-8 framework, together with the APTES-derived silica-like layer, which reduce the direct exposure of CsPbBr_3_ QDs to the external environment.

The fluorescence behavior of CsPbBr_3_@ZIF-8 in different solvents was examined to assess the influence of solvent environment on its emission stability. As shown in Figure 4c, the composite exhibited distinct solvent-dependent fluorescence responses. Detectable emission was observed in dichloromethane, ethanol, toluene, acetonitrile, and n-hexane, whereas the fluorescence signal was almost completely suppressed in DMF and water. The pronounced signal loss in DMF and water indicates that highly polar and coordinating media can adversely affect the emissive CsPbBr_3_ component, probably by disturbing the surface state or structural integrity of the quantum dots. The solvent-dependent shift in the emission maximum may be attributed to differences in solvent polarity and coordinating ability, which alter the local dielectric environment and surface states of the CsPbBr_3_ QDs. Among the tested solvents, ethanol provided a suitable balance between fluorescence response, ENR solubility, solvent compatibility, low toxicity, and applicability to real-sample pretreatment. Therefore, ethanol was selected as the medium for subsequent fluorescence sensing experiments.

The ethanol-phase fluorescence stability of CsPbBr_3_@ZIF-8 was further evaluated under the optimized sensing conditions. As shown in Figure 4d, the emission intensity remained nearly unchanged over 60 min after dispersion, with fluctuations limited to approximately 5% of the initial signal. Such a stable time-dependent profile indicates that ethanol did not induce noticeable fluorescence attenuation during the detection period. This short-term stability is important for quantitative sensing, as it ensures that the fluorescence changes observed in subsequent experiments can be mainly attributed to the interaction between CsPbBr_3_@ZIF-8 and ENR rather than signal drift of the probe itself.

### 3.3. Fluorescence Response Behavior of CsPbBr_3_@ZIF-8 Toward ENR

**Figure 5:**
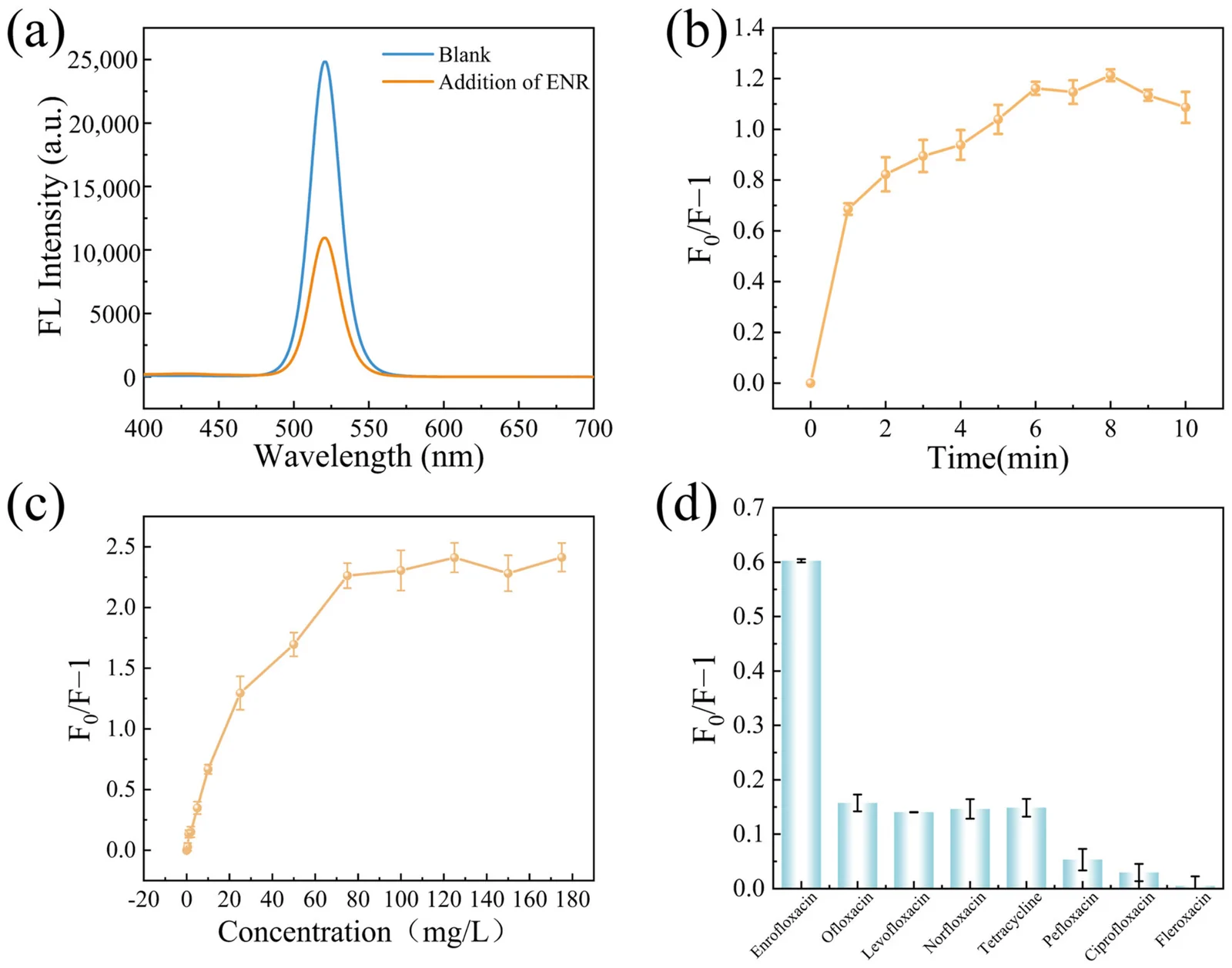
(**a**) Fluorescence emission spectra of CsPbBr_3_@ZIF-8 in the absence and presence of 25 mg·L^−1^ ENR under excitation at 365 nm. (**b**) Dynamic fluorescence response of CsPbBr_3_@ZIF-8 upon addition of 25 mg·L^−1^ ENR. (**c**) Static fluorescence response of CsPbBr_3_@ZIF-8 to increasing ENR concentration. (**d**) Selectivity of CsPbBr_3_@ZIF-8 toward ENR against OFL, LEV, NOR, TC, PEF, CIP, and FLX at 10 mg·L^−1^.

The fluorescence response of CsPbBr_3_@ZIF-8 toward ENR was evaluated in ethanol to assess its feasibility as a responsive fluorescent platform. Figure 5a shows that the CsPbBr_3_@ZIF-8 dispersion emitted green fluorescence under 365 nm UV irradiation, corresponding to a characteristic emission band centered at approximately 520 nm. Upon the introduction of ENR, the visible green emission was substantially attenuated, and the emission intensity at 520 nm decreased accordingly, whereas the peak position remained nearly unchanged. This spectral behavior suggests that ENR mainly reduced the emission intensity of CsPbBr_3_@ZIF-8 without altering its intrinsic emission profile. The clear fluorescence attenuation induced by ENR provides preliminary evidence for the use of CsPbBr_3_@ZIF-8 as a turn-off fluorescent probe for ENR detection.

#### 3.3.1. Dynamic Fluorescence Response

The dynamic fluorescence response of CsPbBr_3_@ZIF-8 toward ENR was investigated to determine a suitable reaction time for fluorescence detection. 25 mg·L^−1^ ENR was added to the CsPbBr_3_@ZIF-8 dispersion, and the quenching efficiency (*F*_0_/*F* − 1) was monitored as a function of reaction time. As shown in Figure 5b, the quenching efficiency increased rapidly within the first 1 min, indicating that ENR could quickly interact with CsPbBr_3_@ZIF-8 and induce an immediate fluorescence quenching response. This rapid initial response may be related to the fast diffusion of ENR molecules in ethanol and their efficient contact with the surface or accessible active sites of CsPbBr_3_@ZIF-8. Subsequently, the quenching efficiency continued to increase slowly from 1 to 6 min, suggesting a gradual enhancement of the interaction between ENR and CsPbBr_3_@ZIF-8. After 6 min, the quenching efficiency remained nearly stable, indicating that the system had reached a relatively steady fluorescence response. Therefore, 6 min was selected as the response time for subsequent fluorescence measurements, which ensured sufficient interaction between ENR and CsPbBr_3_@ZIF-8 while maintaining rapid detection efficiency.

#### 3.3.2. Static Fluorescence Response

The concentration-dependent fluorescence response of CsPbBr_3_@ZIF-8 toward ENR was examined after fixing the response time at 6 min. As shown in Figure 5c, the quenching efficiency (*F*_0_/*F* − 1) increased progressively with ENR concentration, confirming that the fluorescence attenuation of CsPbBr_3_@ZIF-8 was closely associated with the amount of ENR introduced into the system. A pronounced increase was observed at low ENR concentrations, where (*F*_0_/*F* − 1) reached approximately 1.3 at 25 mg·L^−1^, suggesting an efficient quenching response of the probe toward ENR. With further increasing ENR concentration, the response continued to increase and reached approximately 2.2 at 75 mg·L^−1^. Beyond this concentration, the increase became less evident, and the quenching efficiency fluctuated within a narrow range of approximately 2.1–2.4 from 100 to 175 mg·L^−1^. This plateau-like behavior indicates that the fluorescence response gradually approached saturation at relatively high ENR concentrations. Therefore, the concentration-dependent quenching profile demonstrates the responsiveness of CsPbBr_3_@ZIF-8 toward ENR and provides a practical basis for defining the subsequent quantitative detection range.

#### 3.3.3. Selectivity of CsPbBr_3_@ZIF-8 Toward ENR

The selectivity of CsPbBr_3_@ZIF-8 for ENR was evaluated by comparing its fluorescence response with those of other representative antibiotics, including fluoroquinolone analogues such as OFL, LEV, NOR, PEF, CIP, and FLX, as well as TC. As shown in Figure 5d, ENR produced a clearly distinguishable quenching response, with an (*F*_0_/*F* − 1) value of approximately 0.60. By contrast, OFL, LEV, NOR, and TC generated much lower responses, with (*F*_0_/*F* − 1) values of approximately 0.14–0.16, whereas PEF, CIP, and FLX induced only weak fluorescence changes, with values below 0.06. This response pattern shows that the quenching effect was not simply governed by the common fluoroquinolone skeleton, since structurally related antibiotics produced substantially different responses under the same conditions.

The differentiated response may be associated with variations in molecular substituents, electronic distribution, and steric configuration among the tested antibiotics. Previous studies have reported that fluoroquinolone analogues containing similar carboxyl and carbonyl groups can still exhibit different sensing responses because substituent groups influence their interaction strength with fluorescent probes or coordination centers [33]. In this system, the more evident quenching response toward ENR suggests a more favorable interaction between ENR and CsPbBr_3_@ZIF-8 compared with the other tested antibiotics. These results indicate that CsPbBr_3_@ZIF-8 possesses satisfactory selectivity for ENR detection in the presence of common antibiotic analogues. The concentration of 10 mg·L^−1^ used in the selectivity experiment was selected only to compare the relative fluorescence responses of different antibiotics under identical conditions, rather than to represent the regulatory residue level in food matrices.

### 3.4. Fluorescence Quenching Mechanism of CsPbBr_3_@ZIF-8 Toward ENR

**Figure 6:**
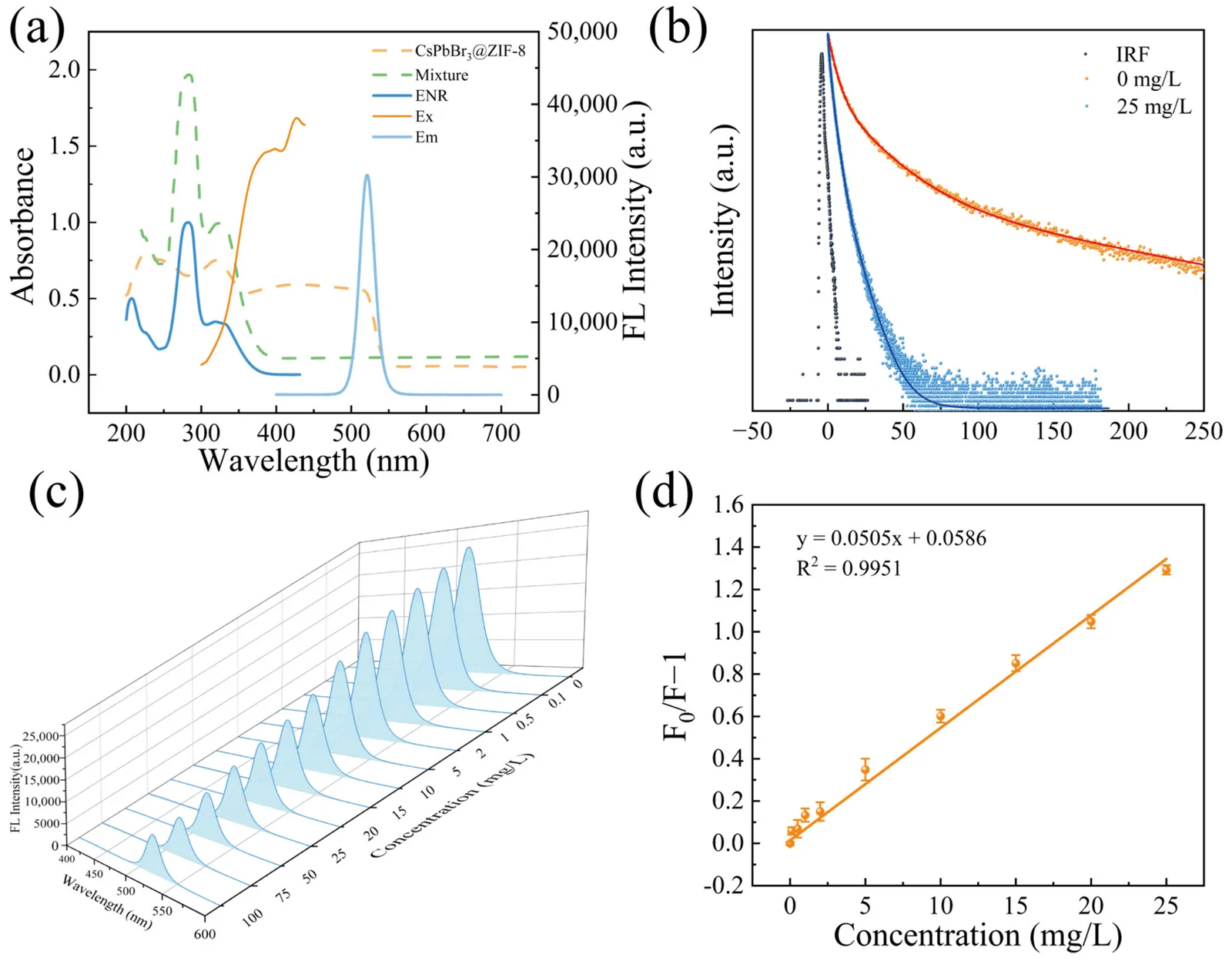
(**a**) UV-vis absorption spectra of ENR CsPbBr_3_@ZIF-8 and mixture, together with the fluorescence excitation and emission spectra of CsPbBr_3_@ZIF-8. (**b**) IRF and fluorescence decay curves of CsPbBr_3_@ZIF-8 in the absence and presence of 25 mg·L^−1^ ENR. (**c**) Fluorescence response spectra of CsPbBr_3_@ZIF-8 toward different ENR concentrations. (**d**) Linear relationship between fluorescence quenching efficiency (*F*_0_/*F* − 1) and ENR concentration.

To clarify the sensing mechanism, possible fluorescence quenching pathways, including intramolecular charge transfer (ICT), inner filter effect (IFE), fluorescence resonance energy transfer (FRET), static quenching, and electron transfer (ET), were investigated. After ENR addition, the emission maximum of CsPbBr_3_@ZIF-8 showed no noticeable displacement, indicating that ICT was not the dominant quenching pathway [34]. As shown in Figure 6a, the UV–vis absorption spectra of CsPbBr_3_@ZIF-8, ENR, and their mixture were recorded at concentrations close to those used in the fluorescence measurements. ENR exhibited absorption mainly in the short-wavelength region, with only weak absorption near the excitation wavelength of 365 nm and negligible absorption near the emission wavelength of 520 nm. Therefore, the secondary inner-filter effect was considered limited, whereas a contribution from the primary inner-filter effect could not be completely excluded. The weak overlap between the ENR absorption spectrum and the emission spectrum of CsPbBr_3_@ZIF-8 also suggested that FRET was unlikely to be the dominant quenching pathway.

Fluorescence lifetime measurements were further performed to distinguish between static and dynamic quenching. The fluorescence decay curves of CsPbBr_3_@ZIF-8 before and after ENR addition were fitted using a tri-exponential decay model (Figure S1):

$$y=A_{1}\times exp\left(-\frac{x}{\tau_{1}}\right)+A_{2}\times exp\left(-\frac{x}{\tau_{2}}\right)+A_{3}\times exp\left(-\frac{x}{\tau_{3}}\right)+y_{0}$$

The average fluorescence lifetime was calculated as follows:

$$\tau_{avg}=\frac{A_{1}{\tau_{1}}^{2}+A_{2}{\tau_{2}}^{2}+A_{3}{\tau_{3}}^{2}}{A_{1}\tau_{1}+A_{2}\tau_{2}+A_{3}\tau_{3}}$$

Fluorescence lifetime measurements were further performed. Both bi-exponential and tri-exponential models were evaluated, and the corresponding fitting results are compared in Figure S1. Considering the heterogeneous nature of the CsPbBr_3_@ZIF-8 composite and its multicomponent decay behavior, the tri-exponential model was adopted for the subsequent lifetime analysis. As shown in Figure 6b, the normalized fluorescence decay profiles of CsPbBr_3_@ZIF-8 before and after ENR addition are shown together with their corresponding tri-exponential fits and the experimentally measured instrument response function (IRF) Fluorescence lifetime analysis showed that the intensity-weighted average lifetime of CsPbBr_3_@ZIF-8 decreased markedly from 87.06 to 5.59 ns after the addition of 25 mg·L^−1^ ENR. This pronounced lifetime shortening indicates accelerated excited-state relaxation and supports the involvement of a dynamic fluorescence-quenching process. Combined with the spectral results, electron transfer may contribute to the ENR-induced fluorescence attenuation.

### 3.5. Development of CsPbBr_3_@ZIF-8 Fluorescence Sensing Method for ENR Determination

The concentration-dependent fluorescence behavior of CsPbBr_3_@ZIF-8 toward ENR was investigated. As shown in Figure 6c, the fluorescence intensity of CsPbBr_3_@ZIF-8 at 520 nm progressively decreased with increasing ENR concentration, indicating a concentration-dependent fluorescence quenching. The quenching response was analyzed using the Stern–Volmer equation, (*F*_0_/*F*) − 1 = K_SV_ *C*, where *F*_0_ and *F* denote the fluorescence intensities of CsPbBr_3_@ZIF-8 in the absence and presence of ENR, respectively, KSV is the Stern–Volmer quenching constant, and C is the ENR concentration. As shown in Figure 6d, a linear relationship was obtained over the ENR concentration range of 0.1–25.0 mg·L^−1^, with the calibration equation y = 0.0505x + 0.0586 and a coefficient of determination (R^2^) of 0.9951.

The limit of detection (LOD), calculated according to the 3 σ/S criterion, was 0.058 mg·L^−1^ in the final ethanolic extract. The limit of quantification (LOQ) was established at 0.10 mg·L^−1^, corresponding to the lowest validated calibration level at which acceptable accuracy and precision were achieved. After accounting for the initial sample amount and final extract volume, the LODs and LOQs were converted to original-sample-basis values of 29 and 50 μg kg^−1^ for honey, 29 and 50 μg L^−1^ for milk, and 11.6 and 20 μg kg^−1^ for fish and shrimp, respectively. For recovery studies and real-sample analysis, the ENR concentrations determined in the final ethanolic extracts were converted to concentrations in the original samples using the corresponding sample preparation factors.

### 3.6. Precision Evaluation of the CsPbBr_3_@ZIF-8 Fluorescence Sensing Method

The precision of the CsPbBr_3_@ZIF-8 fluorescence sensing method was evaluated using 1.0 mg·L^−1^ ENR as the test concentration. Intra-day and inter-day precision tests were performed by repeated measurements within the same day and on different days, respectively (*n* = 9). The precision was assessed based on the quenching efficiency (*F*_0_/*F* − 1) and the corresponding relative standard deviations (RSDs). The mean (*F*_0_/*F* − 1) values for intra-day and inter-day measurements were 0.119 and 0.121, respectively, showing only minor variation between different measurement periods. The RSDs were 3.7% for intra-day precision and 5.1% for inter-day precision, both within an acceptable range for fluorescence-based analytical methods. The low RSD values and comparable mean responses confirm the good reproducibility and precision of the CsPbBr_3_@ZIF-8 fluorescence sensing method for ENR detection.

### 3.7. Fluorescence Sensing Detection of ENR in Real Samples

The practical applicability of the proposed fluorescence sensing method was further evaluated in four food matrices, namely milk, honey, fish, and shrimp, following the sample preparation procedures described in Section 2.5. After pretreatment, ENR was not detected in the unspiked milk and honey samples. In contrast, endogenous ENR concentrations of 4.1 and 14.0 μg kg^−1^ were determined in the fish and shrimp samples, respectively, by HPLC prior to the recovery experiments.

Recovery experiments were subsequently performed at three matrix-specific fortification levels. Milk samples were spiked at 50, 150, and 400 μg L^−1^, whereas honey samples were fortified at 50, 150, and 400 μg kg^−1^. Fish and shrimp samples were fortified at 20, 60, and 160 μg kg^−1^. The pretreated extracts were quantified using the external calibration curve established in ethanol, and the measured concentrations in the final ethanolic extracts were converted to an original-sample basis using the corresponding sample-preparation factors. For fish and shrimp, the endogenous ENR concentrations were subtracted when calculating the recoveries.

As summarized in Table 1, the recoveries obtained for milk were 86.8%, 103.2%, and 91.4%, with RSDs ranging from 2.0% to 4.5%. For honey, the recoveries were 85.2%, 94.9%, and 104.9%, with RSDs of 2.9–3.8%. The recoveries for fish ranged from 97.4% to 106.8%, with RSDs of 4.3–6.0%, whereas those for shrimp ranged from 101.4% to 105.5%, with RSDs of 3.5–6.8%. Across the four food matrices, recoveries ranged from 85.2% to 106.8%, with all RSD values below 7.0%. These results demonstrate the satisfactory accuracy and precision of the proposed method and support its applicability to the quantitative determination of ENR in the tested food matrices after the established sample pretreatment procedures.

To validate the accuracy of the proposed fluorescence sensing method, the ENR contents determined by the CsPbBr_3_@ZIF-8 sensor in perch, crucian carp, and common carp were compared with those obtained using the national standard HPLC method. As summarized in Table 2, the results obtained by the fluorescence method showed no significant difference from those obtained by HPLC (*p* > 0.05, *t*-test), with p values of 0.18, 0.44, and 0.24 for perch, crucian carp, and common carp, respectively. The agreement between the two methods confirms the reliability and accuracy of the CsPbBr_3_@ZIF-8 fluorescence sensing method for ENR determination in real aquatic product samples. For full regulatory validation, further evaluation at lower fortification levels would be beneficial.

To clarify the practical significance of the proposed method, its analytical performance was compared with representative chromatographic and fluorescence-based methods, as summarized in Table 3.

As shown in Table 3, although LC–MS/MS and HPLC-FLD provide higher sensitivity and multicomponent detection capability, the proposed CsPbBr_3_@ZIF-8 fluorescence method avoids chromatographic separation and provides a response within 6 min after sample extraction, making it suitable for rapid targeted screening of ENR in food samples.

**Table 3:** Comparison of representative methods for enrofloxacin determination in food samples.

Method	LOD (mg·L^−1^)	Linear Range (mg·L^−1^)	Instrumental Analysis/Response Time	Ref.
LC–MS/MS	0.00018	0.001–0.100	6 min	[35]
HPLC-FLD	0.00010	0.0003–0.200	NR	[36]
GO–aptamer fluorescence sensor	0.0053	0.00036–0.144	NR	[37]
CsPbBr_3_@ZIF-8 fluorescence sensor	0.058	0.100–25.0	6 min	This work

### 3.8. Comparison with Previously Reported Fluorescent Sensing Methods for ENR Detection

To further evaluate the analytical performance of the proposed CsPbBr_3_@ZIF-8 fluorescence sensing method, a comparison with previously reported fluorescent sensing methods for ENR detection was performed. The key analytical parameters, including LOD, linear range, and response time.

As shown in Table 4, typical fluorescent sensing platforms for ENR detection include carbon dot-based turn-off/on systems, ternary quantum dot sensors, and molecularly imprinted ratiometric sensors. Compared with these reported works, the CsP-bBr_3_@ZIF-8 sensor developed in this work exhibits a wider linear detection range of 0.1–25.0 mg·L^−1^, covering a broader concentration window than the N-CDs-Cu^2+^ system (1.0–15.0 mg·L^−1^) and the MIP-based ratiometric sensor (0.25–4.00 mg·L^−1^). In terms of detection sensitivity, the LOD of 0.058 mg·L^−1^ is superior to most carbon-based and MIP-based fluorescent sensors in the table. Although the AgInS_2_ QDs system shows a lower LOD (0.024 mg·L^−1^), it requires an extremely long incubation time of 150 min, which severely limits its application in rapid on-site detection. In contrast, the pro-posed sensor achieves a fast response within 6 min, which is comparable to the rapid carbon dot system (4 min) and significantly faster than the MIP sensor (12 min) and the AgInS_2_ QDs system. Benefiting from the encapsulation of ZIF-8, the CsPbBr_3_@ZIF-8 sensor also shows improved stability and anti-interference ability, and delivers satisfactory recoveries (85.2–106.8%) in complex matrices including honey, milk, fish, and shrimp samples. These features make the proposed method promising for practical food safety monitoring. Nevertheless, further investigations into long-term operational stability, large-scale preparation, and batch-to-batch reproducibility are still required.

**Table 4:** Comparison of analytical performance of fluorescent sensing methods for ENR detection.

Fluorescent Sensing Material	LOD (mg·L^−1^)	Linear Range (mg·L^−1^)	Response Time (min)	Reference
N-CDs-Cu^2+^	0.16	1.0–15.0	4 min	[38]
AgInS_2_ QDs	0.024	0.3125–20.00	150 min	[39]
Ternary-emission MIP ratiometric sensor	0.134	0.25–4.00	12 min	[40]
CsPbBr_3_@ZIF-8	0.058	0.1–25.0	6 min	This work

### 3.9. Greenness Assessment Results

The environmental performance of the proposed analytical procedure was evaluated using the AGREE metric. As shown in Figure S2, the method obtained an overall AGREE score of 0.42. Favorable contributions were associated with the absence of analyte derivatization and chromatographic separation and the relatively short fluorescence-response step. However, the overall score was limited by the multistep sample-preparation procedure, off-line laboratory analysis, limited automation, organic-solvent consumption, energy-demanding operations, and waste generation. In particular, the use of dichloromethane, acetonitrile, and n-hexane represents an important limitation from the perspective of green analytical chemistry. Therefore, future optimization should focus on reducing solvent consumption, simplifying sample preparation, and replacing hazardous solvents with safer alternatives.

## 4. Conclusions

A stable CsPbBr_3_@ZIF-8 fluorescent sensor was developed for ENR detection by combining ZIF-8-confined growth of CsPbBr_3_ QDs with APTES-assisted surface silanization, thereby improving the ethanol stability of CsPbBr_3_ QDs. The sensor exhibited a linear response to ENR over 0.1-25.0 mg·L^−1^, with a LOD of 0.058 mg·L^−1^. Its applicability was further demonstrated in honey, milk, fish, and shrimp samples, with recoveries ranging from 85.2% to 106.8%. For aquatic product samples, the results obtained by the proposed fluorescence method showed no significant difference from those obtained by HPLC, confirming its reliability for real-sample analysis. This work provides a simple and reliable fluorescence sensing strategy for ENR residue detection and highlights the potential of ZIF-8-protected perovskite quantum dots for food safety monitoring. Further validation of long-term stability and batch-to-batch reproducibility is warranted to support routine application in food monitoring.

## Figures and Tables

**Table 1:** Recovery of ENR from honey, milk, fish, and shrimp samples fortified at different levels.

Sample	Original *	Spiked *	Found *	Recovery (%)	RSD (%, n = 3)
		50.0	43.4	86.8	4.1
Milk	ND	150.0	154.8	103.2	2.0
(μg L^−1^)		400.0	365.4	91.4	4.5
		50.0	42.6	85.2	3.8
Honey	ND	150.0	142.4	94.9	2.9
(μg kg^−1^)		400.0	419.6	104.9	3.7
		20.0	24.5	102.0	6.0
Fish	4.1	60.0	68.2	106.8	4.3
(μg kg^−1^)		160.0	160.0	97.4	5.5
		20.0	35.1	105.5	6.8
Shrimp	14.0	60.0	75.1	101.8	4.4
(μg kg^−1^)		160.0	176.3	101.4	3.5

* Concentrations are expressed in μg L^−1^ for milk and in μg kg^−1^ for honey, fish, and shrimp. ND, not detected.

**Table 2:** Comparison of HPLC and the proposed fluorescence sensing method for ENR determination in aquatic products.

Samples	CsPbBr_3_@ZIF-8 Sensor (μg kg^−1^, ±SD)	HPLC Method (μg kg^−1^, ±SD)	*p*
Perch	65.1 ± 0.5	64.3 ± 0.7	0.18
Crucian carp	76.6 ± 0.8	77.0 ± 0.4	0.44
Common carp	21.1 ± 0.5	20.1 ± 0.7	0.24

## Data Availability

The original contributions presented in this study are included in the article/Supplementary Materials. Further inquiries can be directed to the corresponding author.

## Supplementary Materials

The following supporting information can be downloaded at https://www.mdpi.com/article/10.3390/foods15162772/s1. Method S1. HPLC reference analysis of enrofloxacin in aquatic product samples; Figure S1: Fluorescence lifetime decay curves of CsPbBr_3_@ZIF-8 fitted with double-exponential model (left) and tri-exponential model (right); Figure S2: Greenness assessment of the proposed analytical procedure using the AGREE metric.
