# Supplementary material for: ZIF-8-Confined and APTES-Passivated CsPbBr3 Quantum Dots for Fluorescence Detection of Enrofloxacin Residues in Foods
Source: Foods. 2026 Aug 7;15(16):2772. doi: 10.3390/foods15162772 (PMC13511832; doi:10.3390/foods15162772)
Supplement: Supplementary file 1 [file foods-15-02772-s001.zip › foods-4415609-supplementary.pdf]

ZIF-8-Confined and APTES-Passivated $CsPbBr_3$ Quantum Dots for Fluorescence Detection of Enrofloxacin Residues in Foods

Guanru Yi, Shaoyu Lv, Longwei Fu, Zhixiang Xu, Shiyong Wang and Longhua Xu *

College of Food Science and Engineering, Shandong Agricultural University, Tai'an 271018, China; yi784904607@163.com (G.Y.); lvshaoyu2021@163.com (S.L.); 18863015339@163.com (L.F.); zhixiangxu@sina.com (Z.X.); hzwangshiyong@163.com (S.W.)

* Correspondence: longhuaxu@sdau.edu.cn

Method S1. HPLC reference analysis of enrofloxacin in aquatic product samples

S1.1. Sample preparation

Aquatic product samples were pretreated with reference to the Chinese National Food Safety Standard GB 31656.3-2025. Briefly, 5.00 ± 0.02 g of homogenized edible tissue was accurately weighed into a centrifuge tube. Acidified acetonitrile solution (20 mL) and anhydrous sodium sulfate (10 g) were added, and the mixture was homogenized at high speed for 1–2 min. After centrifugation at 3000 rpm for 5 min, the supernatant was transferred into a separatory funnel. The homogenizer head and sample residue were washed and re-extracted twice, each time using 20 mL of acidified acetonitrile. All supernatants were combined.

Subsequently, 60 mL of acetonitrile-saturated n-hexane was added to the combined extract. The mixture was shaken for 20 min and allowed to stand for phase separation. The lower acetonitrile phase was collected in a pear-shaped flask and evaporated to dryness under reduced pressure at 40 °C.

The dried residue was reconstituted with 4 mL of phosphate buffer, sonicated for 1 min, allowed to stand for 30 s, and transferred into a 15 mL centrifuge tube. The residual material in the flask was reconstituted twice more using the same procedure. The three reconstitution solutions were combined and centrifuged at 3000 rpm for 5 min, and the resulting supernatant was collected for solid-phase extraction cleanup.

A C18 solid-phase extraction cartridge was sequentially conditioned with 3 mL of methanol, 3 mL of water, and 3 mL of phosphate buffer. The collected supernatant was loaded onto the cartridge at a flow rate of approximately one drop per second. The cartridge was then washed with 3 mL of water and dried under vacuum. The analytes were eluted with 5.0 mL of elution solution. The collected eluate was evaporated to dryness under a nitrogen stream at 50 °C. The residue was reconstituted in 1.0 mL of the mobile phase and filtered through a 0.22 μm membrane filter before HPLC analysis. The acidified acetonitrile, phosphate buffer, and elution solution were prepared according to GB 31656.3-2021.

2. Results

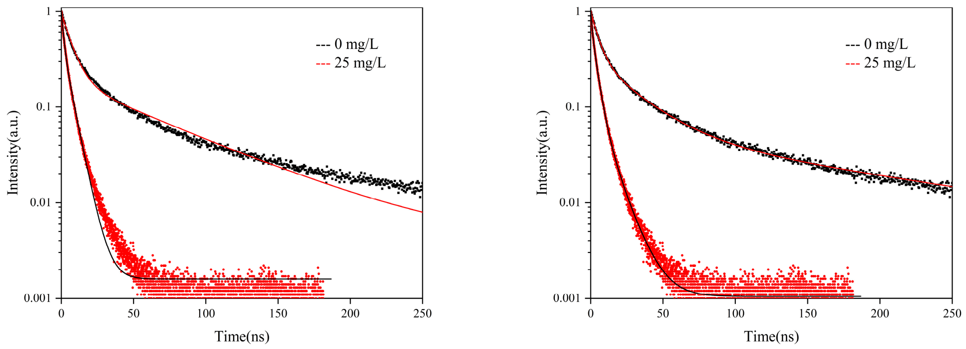

Figure S1. Fluorescence lifetime decay curves of $CsPbBr_3$@ZIF-8 fitted with double-exponential model (left) and tri-exponential model (right).

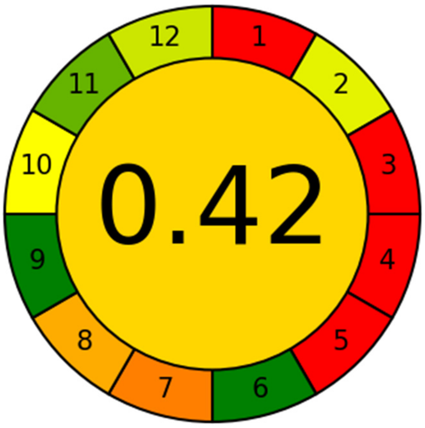

Figure S2. Greenness assessment of the proposed analytical procedure using the AGREE metric.
